# Metagenome-Assembled Genome of *Halomonas* sp. Isolate SL48-SHIP-3 from the Microbial Mat of Salt Lake Number 48 (Novosibirsk Region, Russia)

**DOI:** 10.1128/MRA.01293-19

**Published:** 2019-12-05

**Authors:** Aleksandra A. Shipova, Anton V. Korzhuk, Valeria Shlyahtun, Alla V. Bryanskaya, Aleksey S. Rozanov, Sergey E. Peltek

**Affiliations:** a Federal Research Center–Institute of Cytology and Genetics of the Siberian Branch of the RAS, Novosibirsk, Russia; Georgia Institute of Technology

## Abstract

The Halomonas sp. isolate SL48-SHIP-3 genome was obtained from metagenomics sequencing of the microbial mat of Salt Lake Number 48 (54.201806N, 78.179194E; Novosibirsk region, Russia). The sequenced and annotated genome is 2,575,909 bp and encodes 2,368 genes.

## ANNOUNCEMENT

Halomonas is one of the genera of the family Halomonadaceae (Proteobacteria). These bacteria are halophiles that were previously discovered in other regions of the planet, including salt lakes (1).

Salt Lake Number 48 (54.201806N, 78.179194E; Novosibirsk Region, Russia) is a chloride sulfate lake (pH 8.0), with salinity ranging from 190 g/liter to 230 g/liter depending on weather conditions (2). A sample of the microbial mat, which consists of floating particles 2 to 10 mm in size, was taken from the coastal part of this lake and stored in alcohol at −70°C. From this sample, a metagenome was obtained, from which the genomes of individual microorganisms were then isolated. We have already described one of the genomes in a previous article (3). Total DNA was isolated using the NucleoSpin soil kit (genomic DNA from soil) with the default protocol. Libraries for metagenome sequencing were prepared at the Center of Genomic Studies, Institute of Cytology and Genetics of the Siberian Branch of the RAS (ICG SB RAS), using a NEBNext Ultra DNA library prep kit for Illumina; the insert size was 450 bp. Metagenome paired-end sequencing was performed on a NovaSeq platform (Illumina) at Genetico LLC using the NovaSeq 6000 S2 reagent kit (200 cycles).

A total of 398,852,702 reads were sequenced; the average read length was 100 bp. The reads were processed by Trimmomatic version 0.36 (using options MINLEN:95 and CROP:97) (4). *De novo* assembly of short reads into scaffolds was performed using SPAdes version 3.11.1 with the option “-only-assembler” (5). Contigs shorter than 1,000 bp were deleted. Binning of metagenomics scaffolds into separate clusters (in which one bin represents one genome) was carried out using MaxBin (version 2.2.4) with default parameters (6).

Here, we describe one of the resulting genomes with coverage of 629.5×. The classification of this genome to the family level was obtained using MetaWRAP version 1.2.2 (7) with the option “classify_bins.” The genome belongs to the family *Halomonadaceae* (within the class Gammaproteobacteria). We aligned amino acid sequences (which were found by MaxBin for this cluster) against the NCBI nr protein database. Most of them had the best match (with the highest score and an E value of <0.05) with *Halomonas* sp. strain es.049. We compared this genome with the reference genomes of the most closely related members of the genus *Halomonas* (that had a match with the described genome) using the Average Nucleotide Identity (ANI) calculator (http://enve-omics.ce.gatech.edu/ani/) with default parameters. The genomes of Halomonas arcis strain CGMCC (identity, 77.78%), Halomonas campaniensis strain LS21 (identity, 78.04%), and *Halomonas* sp. es.049 (identity, 80.18%) were closest to our genome. These results suggest that our organism may constitute a new strain. The genome was checked for contamination using CheckM version 1.0.13 (8) (with the option “taxonomy_wf family Halomonadaceae”); the contamination rate was 0.64%, and the completeness rate was 92.18%.

The genome is 2,575,909 bp long, consists of 149 contigs, and has a GC content of 56.79% and an *N*_50_ value of 37,322 bp (evaluated using QUAST version 5.0.2 with default parameters [9]). Open reading frame (ORF) prediction and automatic annotation were performed using NCBI PGAP (version 4.8) with the default parameters (10). The complete genome sequence contains 2,446 genes, 2,388 coding sequences (CDS), 1 rRNA (23S), 53 tRNAs, and 4 noncoding RNAs (ncRNAs).

### Data availability.

The raw metagenomics data have been deposited at DDBJ/EMBL/GenBank under accession no. SRR7943696. The draft genome sequence for *Halomonas* sp. isolate SL48-SHIP-3 has been deposited at DDBJ/EMBL/GenBank under accession no. VMQS00000000. The 149 contigs have been deposited under accession no. VMQS01000001 to VMQS01000149 (https://www.ncbi.nlm.nih.gov/Traces/wgs/VMQS01?display=contigs).
